# Investigations on the Mechanical Properties of Glass Fiber/Sisal Fiber/Chitosan Reinforced Hybrid Polymer Sandwich Composite Scaffolds for Bone Fracture Fixation Applications

**DOI:** 10.3390/polym12071501

**Published:** 2020-07-06

**Authors:** Soundhar Arumugam, Jayakrishna Kandasamy, Ain Umaira Md Shah, Mohamed Thariq Hameed Sultan, Syafiqah Nur Azrie Safri, Mohd Shukry Abdul Majid, Adi Azriff Basri, Faizal Mustapha

**Affiliations:** 1 School of Mechanical Engineering, Vellore Institute of Technology, Vellore 632014, India; soundhar1372214@gmail.com; 2 Laboratory of Biocomposite Technology, Institute of Tropical Forestry and Forest Products (INTROP), Universiti Putra Malaysia, UPM Serdang 43400, Selangor Darul Ehsan, Malaysia; ainumaira91@gmail.com (A.U.M.S.); snasafri@gmail.com (S.N.A.S.); 3 Department of Aerospace Engineering, Faculty of Engineering, Universiti Putra Malaysia, UPM Serdang 43400, Selangor Darul Ehsan, Malaysia; adiazriff@upm.edu.my (A.A.B.); faizalms@upm.edu.my (F.M.); 4 Aerospace Malaysia Innovation Centre (944751-A), Prime Minister’s Department, MIGHT Partnership Hub, Jalan Impact, Cyberjaya 63000, Selangor Darul Ehsan, Malaysia; 5 School of Mechatronic Engineering, Pauh Putra Campus, Universiti Malaysia Perlis, Arau, Perlis 02600, Malaysia; shukry@unimap.edu.my

**Keywords:** sandwich composites, glass fiber, chitosan, mechanical properties, water absorption, porosity, bone plate

## Abstract

This study aims to explore the mechanical properties of hybrid glass fiber (GF)/sisal fiber (SF)/chitosan (CTS) composite material for orthopedic long bone plate applications. The GF/SF/CTS hybrid composite possesses a unique sandwich structure and comprises GF/CTS/epoxy as the external layers and SF/CTS/epoxy as the inner layers. The composite plate resembles the human bone structure (spongy internal cancellous matrix and rigid external cortical). The mechanical properties of the prepared hybrid sandwich composites samples were evaluated using tensile, flexural, micro hardness, and compression tests. The scanning electron microscopic (SEM) images were studied to analyze the failure mechanism of these composite samples. Besides, contact angle (CA) and water absorption tests were conducted using the sessile drop method to examine the wettability properties of the SF/CTS/epoxy and GF/SF/CTS/epoxy composites. Additionally, the porosity of the GF/SF/CTS composite scaffold samples were determined by using the ethanol infiltration method. The mechanical test results show that the GF/SF/CTS hybrid composites exhibit the bending strength of 343 MPa, ultimate tensile strength of 146 MPa, and compressive strength of 380 MPa with higher Young’s modulus in the bending tests (21.56 GPa) compared to the tensile (6646 MPa) and compressive modulus (2046 MPa). Wettability study results reveal that the GF/SF/CTS composite scaffolds were hydrophobic (CA = 92.41° ± 1.71°) with less water absorption of 3.436% compared to the SF/CTS composites (6.953%). The SF/CTS composites show a hydrophilic character (CA = 54.28° ± 3.06°). The experimental tests prove that the GF/SF/CTS hybrid composite can be used for orthopedic bone fracture plate applications in future.

## 1. Introduction

Bone plate is used to arrest the bone fracture and lessen the fracture gap, thereby enabling primary bone healing. The primary part of bone plate is to locate the fractured bone in a correct location and avoid tensile stresses at the fractured surface [1]. High stiffness titanium and stainless-steel alloys are often utilized for bone plate applications owing to their properties such as mechanical strength, corrosion resistance, bio-inertness, and cost effectiveness compared to other biomaterials [2]. However, this clinically available fixation of long bone fracture metal plate is less preferred because of its stiffness mismatch between the metal plate and human cortical bone, which is commonly referred as stress shielding effect [3]. Due to the stiffness mismatch, more load transfer can be observed at the bone plate instead of the cortical bone, thereby distracting the vascularity of the bone beneath the plate, causing bone resorption and diminution in its strength on a longer duration [4]. Therefore, it is essential to minimize the destructive effect of stress shielding by designing a matching fracture plate to the mechanical properties of the cortical bone. The stress shielding effect can be minimized by using composite materials as a replacement to titanium and stainless-steel alloy-based orthopedic implants [5]. 

Nowadays, many classes of polymers are utilized in all fields of medicine [6,7]. The availability of various polymer types with personalized formulations such as accustomed blends, degree of crystallization, molecular weight, crosslinking degree, additional bioactive surface functionalization, copolymers, and permit this broad variety of medical applications [8]. The selection of a polymer is primarily based on its engineering properties like elasticity, tensile strength, stiffness, and other related properties such as biocompatibility and toxicity [9]. Polymer-based materials such as poly (hydroxyl butyrate) (PHB), poly (lactic acid) (PLA), poly (glycolic acid) (PGA), and poly (ether ether ketone) (PEEK) are utilized for orthopedic applications [10,11]. The core problem related with the use of polymers are inadequate tribological wear and other mechanical properties; it can be rectified by adding synthetic fibers into the polymer matrix to improve the mechanical and wear properties [12]. Suner et al. (2013) compared the volume and size distributions, wear rate, biocompatibility, and bioactivity of the wear debris produced from Multi-Walled Carbon Nanotube (MWCNT) nanocomposite in comparison with the traditional ultra-high molecular weight polyethylene (UHMWPE). The wear rate of composite was significantly reduced by adding MWCNT. The results prove that the UHMWPE/MWCNT nanocomposites are a better replacement for orthopedic applications [13]. 

In recent years, knitted and braided fabric reinforced composites have been fabricated to encounter the biological and mechanical requirements for efficient bone healing. The mechanical characterization of braided composites can be modified by changing the parameters like fiber volume fraction, stacking angle, braid angle, and fabrication method [14]. Fujihara et al. (2004) manufactured compression bone plates made up of braided laminated composites, which were surgically used to repair bones with diaphyseal fracture. The fatigue and static bending properties of braided laminates were studied. Various braiding angles (15°, 20°, and 25°) of braided fabric reinforcement were examined in the view of microstructure. The experimental data prove that at the smallest braiding angle of 15°, the static bending property of the composite was higher, while the healthy fracture resistance under fatigue load was obtained at 20° braiding angle [15]. Li et al. (2017) prepared the composite with matrix of PLA and reinforcement of two-dimensional (2D)-braided magnesium (Mg) wires for orthopedic implant applications. The highest shear and impact strengths were obtained at a 45° braiding angle. As the volume fraction increases, the bending, shear, impact and tensile strengths also increases [16]. Zhao et al. (2017) investigated the bio mineralization and “in vitro” degradation of PLA composite reinforced with Mg particles using the solvent casting method. The study explicated that the affirmative effects of Mg combined to PLA matrix on the osteogenesis and degradation, and provided a substitute for the presently used PLA implants [17]. Wan et al. (2007) examined the composite prepared by hybridizing kevlar fibers and three-dimensional (3D)-braided carbon as a reinforcement with bismaleimide (BMI) resin. They concluded that the synergic effect between the kevlar fiber and braided carbon fiber contributed to the increase in flexural and shear strength of hybrid composites (3D-braided kevlar/BMI/carbon) [18]. However, their success depends on the biocompatibility of the matrix and fibers. Again, with respect to the matrix, epoxy is one of the most broadly utilized polymers in orthopedic and dentistry applications [19]. 

Natural fibers are preferred for their low density, high stiffness-to-weight ratio, low cost, high strength-to-weight ratio, and eco-friendly characteristics [20]. Recently, researchers are working to identify potential plant fibers for medium- and low-load applications by characterizing their mechanical properties. The favorable woven natural fabric composites properties have increased their usage in biomedical applications [21]. Pothan et al. (2008) related the mechanical properties of woven sisal fiber (SF) composite and established that the usage of plain-woven fabric improves the composite properties [22]. Shibata et al. (2008) found that the flexural strength of random and unidirectional oriented bamboo/kenaf fiber reinforced composites, and inferred that, irrespective of any fiber type, the woven fabric enhances the flexural modulus and composite strength [23].

However, the limitations of natural fibers are hydrophilicity and moisture absorption. In general, the outer surface of the bone plate has more contact with human cells, tissues and other biological fluids. This contact increases the wettability and affects the surface properties of the material used. Researchers have suggested that blood-contacting devices, bone plates, and tissue engineering substrates must possess the stability of hydrophobic and hydrophilic characteristics for an enhanced biocompatibility [24,25]. Hybrid composites (a mixture of two or more synthetic and natural fibers into a single common matrix) are preferred to overcome the limitations of natural fibers [26]. Khanam et al. (2010) conducted an experiment on hybrid composites reinforced with SF/carbon fiber and reported that the inclusion of carbon fiber enhances the flexural and tensile properties of hybrid composites [27]. Rajesh and Pitchaimani (2016) studied the influence of fiber orientations and fiber yarn on mechanical characteristics such as flexural, impact, and tensile properties. They found that the natural fiber-braided yarn offers excellent mechanical properties compared to the conventional woven fabric and concluded that the natural fiber in random and short fiber offers poor mechanical strength [28]. Sathishkumar et al. (2017) examined the polyester matrix reinforced with cotton/SF hybrid composites for structural applications. It was concluded that a 40% volume fraction of the cotton/SF-reinforced polyester hybrid composites exhibit good vibration characteristics and mechanical properties [29]. Romanzini et al. (2013) explained the influences of glass fiber (GF) and ramie natural fiber on dynamic mechanical behavior, and found that the addition of GF improves the stiffness of the composite [30]. Ahmed and Vijayarangan (2008) blended woven jute fiber with woven GF composites for tensile and flexural strength. They observed that the mechanical properties of the sandwich composite improved when the GF was kept as an outer layer [31]. Similar variations were observed by Jarukumjorn and Suppakarn (2009) for GF/SF hybrid composites and noticed that the synergic effect enhances the composite mechanical properties [32]. Gouda et al. (2014) prepared a hybrid natural-fiber polymer composite made up of two different percentages (16% and 24%) of epoxy with banana, SF, hemp, and jute fibers and compared their mechanical and physical properties against the strength of a femur bone. The experimental results confirmed that the hybrid composite with 24 wt% of hybrid natural fibers show better mechanical properties compared to the hybrid composite with 16 wt% of the hybrid natural-fiber composite [33]. Manteghi et al. (2017) also proposed flax/GF/epoxy sandwich hybrid composite for the fixation plates of bone fracture [34]. 

Few researchers have also studied the influence of filler materials on hybrid composites. Yunus and Alsoufi (2018) prepared high-density polyethylene matrix composites with addition of bio-ceramic materials such as alumina (Al_2_O_3_) and titanium oxide (TiO_2_) for orthopedic applications (bone fracture plate, bone cement, bone graft, and hip joint replacement) [35]. Manjubala et al. (2018) prepared hydroxyapatite (HA)/carboxyl methyl cellulose (CMC) composites for bone regeneration applications. This study suggested that HA/CMC composite scaffolds are suitable for the bone tissue engineering field due to their mechanical stability, biocompatibility, and biodegradable properties [36]. Chitosan (CTS), also known as (1–4)-linked 2-amino-2-deoxy-b-glucan, is a by-product of N-deacetylation of chitin. It is generally obtained from shrimp shells, insect cuticles, and crab shells [37]. Researchers have investigated the feasibility of utilizing CTS and chitin as novel biomaterials. CTS is also widely applied in biomedical fields such as wound healing, artificial organs, gene delivery, biosensors, antimicrobials, and scaffolds for tissue regeneration due of its biocompatibility, hemocompatibility, cytocompatibility, and biodegradable properties [38]. Amri et al. (2013) discussed the mechanical and thermal properties of polypropylene composites reinforced with CTS fiber and it was concluded that addition of CTS improved the Young’s modulus, thermal stability, and impact strength of composites [39]. Li et al. (2019) investigated the bio functionalization of biomaterials with synthetic Wnt5a mimetic ligand (Foxy5 peptide) to enhance the osteogenesis and mechanosensing of human mesenchymal stem cells by stimulating noncanonical Wnt signaling. They found that immobilized Wnt5a mimetic ligand-activated noncanonical Wnt signaling leading to enhanced intracellular calcium level, F-actin stability, actomyosin contractility, and cell adhesion structure development [40]. Kang et al. (2019) suggested that macrophages play a significant role in regulating foreign body response in biomaterials. The macrophages in biomaterials can reversibly convert their cell adhesion and promote the tissue healing processes by regulating the inflammatory of tissues [41]. Bagheri et al. (2015) prepared CF/Flax/Epoxy composite plate for bone fracture plate to be used in orthopedic trauma applications. They found that CF/Flax/Epoxy composite plate showed similar cell viability with no adverse effect on gene expression levels observed for bone formation in comparison to medical-grade stainless steel. The study results recommended that the combination of synthetic/natural fiber may have the potential for bone plate applications [42]. Soundhar and Jayakrishna (2019) fabricated CTS nanoparticles reinforced epoxy polymer composites for bone plate applications. They found that the addition of CTS up to 4 wt% enhanced the flexural (47%) and tensile strength (80%). They also observed that the hemolytic ratio was less than 5% at 4 wt% of CTS, which makes it suitable for orthopedic applications [43]. Table 1 shows the mechanical characteristics and application of hybrid polymer composites in details.

Based on the previous literature, only limited studies were conducted on hybrid composites such as carbon fiber/flax/epoxy, GF/flax/epoxy, and roselle/banana/SF for bone plate fracture fixation applications [42,45]. In this work, a hybrid GF/SF/epoxy sandwich composite with CTS as the nanofiller material was developed for orthopedic bone plate application. The bone plate developed is a sandwich structure with the top and bottom layers made up of GF/epoxy and inner layer with SF/epoxy/CTS nanoparticles. 

This study aims to identify the mechanical properties of sandwich hybrid GF/SF/CTS epoxy plate to develop a new kind of biomaterials for orthopedic long bone fracture reparation by enhancing the osteointegration between the bone and hybrid composite scaffolds. The mechanical properties of the newly fabricated composite scaffolds were evaluated through the flexural/bending, compression, tensile, and micro hardness. Scanning electron microscopic (SEM) images were studied to analyze the microstructure of the newly developed composite. Water absorption test was also conducted to identify the quantity of water absorbed under different circumstances. 

## 2. Materials and Methods 

### 2.1. Materials

In this experiment, a GF-woven mat and SF plain-woven mats were used as the primary reinforcements and CTS was utilized as the secondary strengthening in the epoxy matrix. A high molecular weight of CTS (345,500 g·mol^−1^) was procured from M/s India Sea Foods, Kochi, Kerala, India. CTS was obtained by deacetylation of chitin at a deacetylation rate of 85%. The GF-woven mats were purchased from M/s Go Green products, Chennai, India. The SF-woven mats were procured from Jolly enterprise, Kolkata, West Bengal, India. Bisphenol A epoxy resin and hardener (HY951) were acquired from Seenu & Co, Chennai, India. 

### 2.2. Composite Scaffold Preparation

**Figure 1:**
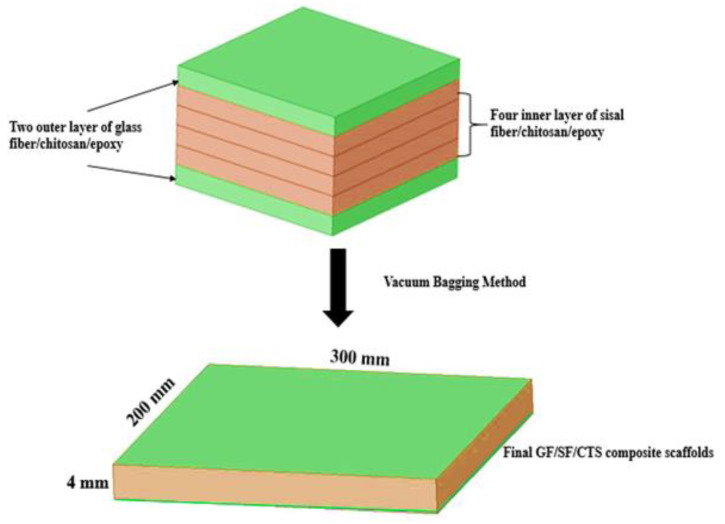
Sandwich structure of GF/SF/CTS composite scaffolds.

The hybrid GF/SF/CTS reinforced epoxy composite scaffolds were prepared using three/four core layers of SF/CTS/epoxy laminates and two layers of GF/SF/CTS laminates as the outer layers of the scaffolds, resulting in a sandwich structure (Figure 1). Primarily, a known amount of epoxy and CTS particles of various weight percentages (0, 1, and 2 wt%) were mixed for 60 min using an ultrasonic sonicator to obtain a homogeneous mixture. The CTS/epoxy mixture was further added to the hardener and the resultant mixture was mechanically stimulated for 10 min.

The mixed resin was poured over the bottom layer of GF, and the inner layer of SF were stacked over the GF; then, the top coat of GF/epoxy was placed above the SF to form the sandwich structure of the composite scaffolds. To remove the excess quantity of resin on the woven fabric, the composite scaffolds were placed inside the vacuum bag at ambient temperature. The hybrid laminates were then detached from the mold after 24 h. This composite plate possesses a unique sandwich structure with a stiff outer layer of synthetic fibers (GF) and inner layers of flexible natural fibers (SF), imitating the overall structure of human bone (spongy internal cancellous and rigid external cortical matrix). Soundhar and Jayakrishna (2019) reported that the addition of chitosan in SF-reinforced polymer composites enhances the mechanical as well as biocompatibility nature of the composites [25]. Therefore, in this study the authors have attempted to utilize the chitosan as a filler material in GF/SF/Epoxy hybrid composites for bone fracture plate applications. The experimental design was developed based on two varying parameters, i.e., the number of layers of sisal fibers and weight percentage of chitosan with constant two outer layers of a glass fiber stacking sequence. The design and composition of the fabricated sample composites noted as A1, A2, A3, A4, A5, and A6 are listed in Table 2.

## 3. Characterizations

### 3.1. Mechanical Properties

Mechanical strength like flexural, tensile, and compressive properties of hybrid GF/SF/CTS composite scaffolds were analyzed using Instron 8801 universal testing machine (UTM). The tensile properties were measured at a crosshead speed of 1 mm/min on the samples of 165 × 25 × 3 mm as per ASTM D 638 standards. The flexural test was conducted at a crosshead speed of 2 mm/min on the samples of 128 × 12.7 × 3 mm as per ASTM D 790 standards [46]. The compressive mechanical properties of composite scaffolds were measured at a crosshead speed of 1 mm/min using Instron (8801) UTM with a 10 kN load as per ASTM D 695 [47]. For confirmation of the results, three values of tensile, flexural and compressive specimens were measured for each stacking sequence and the mean values are calculated. Stress–strain curves for all the mechanical properties presented in this study were the best curves showing consistent repeatability. 

### 3.2. Micro Hardness

The micro hardness measurements were performed at room temperature using a Shimadzu HMV-2 hardness tester with a diamond cone indenter with a 0.2-mm tip radius, at a load of 250 N. The test was performed as per ASTM E384 standard for micro-indentation hardness of materials [48]. A load of 250 N was applied for 10 s on each indentation. For each of the six types of GF/SF/CTS composite scaffolds, 10 readings were recorded, and the average value was recorded as the hardness of the samples.

### 3.3. Scanning Electron Microscopy

The morphology behavior of hybrid GF/SF/CTS composite scaffolds were examined using SEM (Carl ZeissEVO18 instrument, Jena, Germany). Initially, the composite scaffold samples coated with gold were prepared by a sputtering machine for 10 min under a high-vacuum medium.

### 3.4. Moisture Absorption and Wettability

The water absorption property was evaluated by soaking the GF/SF/CTS composite scaffolds in distilled water at ambient temperature as per ASTM D5229 standard to test the moisture absorption properties of composite with the polymer matrix [49]. The hybrid GF/SF/CTS composite scaffold samples were examined using the sessile drop process to determine their hydrophobic and hydrophilic nature. A drop of distilled water was slowly released on the scaffold surface and images were captured through a digital camera. The contact angle (CA) between the surface of scaffold and water drops were then measured. The CA of more than 90° shows a hydrophobic nature and the CA of less than 90° indicates a hydrophilic nature [50].

### 3.5. Porosity of the Composite Scaffolds

The porosity of the GF/SF/CTS composite scaffold samples were determined by using the ethanol infiltration process. Initially, the mass of dry composite scaffolds, *W_i_*, was measured and then the composite scaffolds were immersed in an ethanol medium. After 24 h, the scaffolds were removed from the ethanol medium, and the ethanol over the surface was wiped and the final weight, *W_e_*, was measured quickly. The porosity of the composite was calculated using Equation (1) [51]:

(1) $$\%\;porosity=\frac{W_{e}-W_{i}}{\rho V}\times 100$$

where *ρ* denotes the ethanol density at room temperature, i.e., 0.789 g/mL, and *V* is the volume of the composite scaffold.

### 3.6. Statistical Analysis

The experimental test results of tensile, flexural, compressive strength and modulus values were evaluated in triplicates and expressed in terms of mean ± S.D. Results were analyzed by Two-way ANOVA followed by Unpaired *t*-test using GraphPad Prism 8.4.2 software program (GraphPad Software, San Diego, CA, USA) and the statistical difference was observed to be extremely significant at *p*-value ≤ 0.0001 (****) and *p*-value ≤ 0.001 (***).

## 4. Results and Discussion

### 4.1. Tensile Properties of the Composite Scaffolds

The tensile properties of composite scaffolds were analyzed to understand the influence of the number of SF core layer and inclusion of CTS particles of the hybrid GF/SF/CTS sandwich composites. The mechanical properties of GF/SF/CTS composite scaffolds for the six samples are listed in Table 3. 

**Figure 2:**
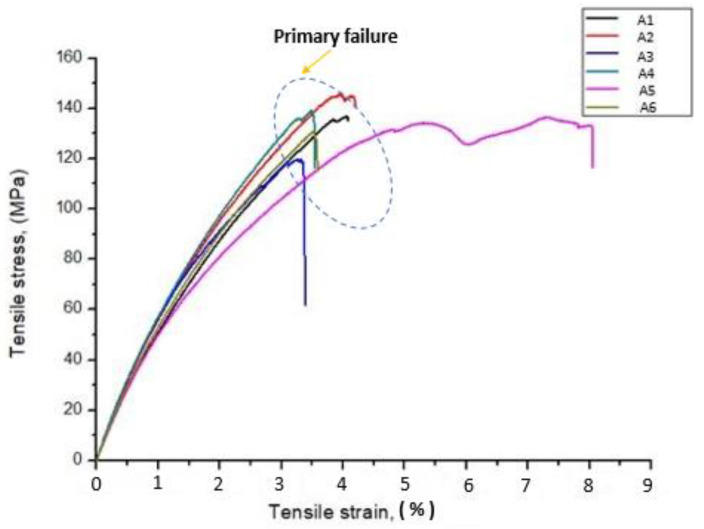
Stress Vs. strain variation of GF/SF/CTS composite scaffolds under tensile test.

The tensile behavior of the stress–strain curve in relation to the composites is shown in Figure 2. The figure shows that the A2 composites have more strength compared to the other composites, due to the presence of more SF layers in the composite scaffolds supporting high loads which enhanced the tensile strength of A2 composite scaffolds. At the same time, A3, A4, A5, and A6 composites have lower tensile strength compared to the A1 and A2 composites, due to the non-uniform dispersal and agglomeration of CTS in SF and GF interface. This interface makes the A3, A4, A5, and A6 composites into an amorphous structure that leads to a premature failure even at low strain rate [43].

**Figure 3:**
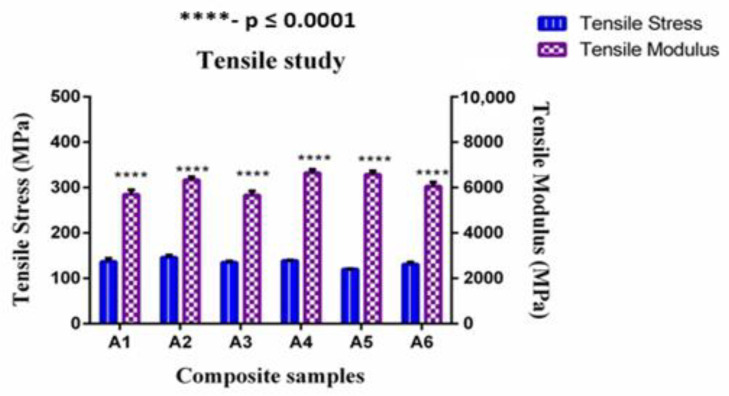
Tensile modulus vs. tensile strength of GF/SF/CTS composite scaffolds. Data are represented as means of triplicate (n = 3) ± SD, where **** indicates *p* ≤ 0.0001.

The influences of reinforcement on the tensile strength of hybrid GF/SF/CTS composites are shown in Figure 3. The composite scaffolds A2 and A4 have higher tensile strength compared to other composite combinations. This is due to the virtuous bond between GF and sisal natural fibers. The addition of CTS up to 1 wt% improved the tensile properties. The inclusion of more than 1 wt% of CTS loadings in three- and four-layer SF-reinforced composite scaffolds decreased the tensile value because of the non-uniform distribution of CTS in the composite scaffolds. The hybridization of three-layer SF and GF improved the tensile strength of A1 and A3 composite scaffolds, while it reduced the tensile strength of A5 composite scaffolds due to higher loading of CTS particles. The tensile strength of the three-core layer of SF in A1 and A3 composites were 14.2% and 12.5% higher than that of A5 composites, respectively. Similar trends were observed in A2, A4, and A6 composites. The tensile strength of four core SF layer samples such as A2 and A4 were 11.5% and 6.3% higher than that of A6 composites.

From the statistical analysis of tensile modulus and tensile strength (Refer Figure 3), it is observed that the sandwich composite scaffolds with four-layer SF (A2, A4) and CTS (0, 1, and 2 wt%) enhances the load-carrying capacity with significant difference (****—*p* value ≤ 0.0001) than the three-layer core sandwich composite scaffolds (A1, A3), which is due to the number of layers in the core. Therefore, it can be inferred that, as the number of layer increases, the load carrying capacity increases from the matrix to fiber, resulting in high modulus of rigidity [52].

### 4.2. Flexural Properties of the Composite Scaffolds

**Figure 4:**
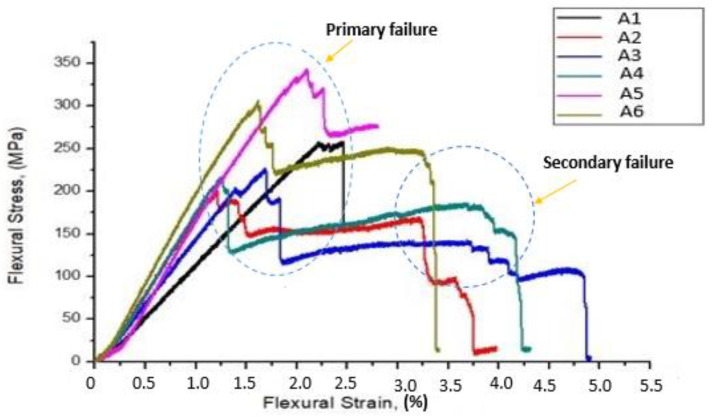
Stress vs. strain variation of GF/SF/CTS composite scaffolds under a three-point bending test.

The flexural test was conducted on the composite scaffolds with the span of 90 mm and span–thickness ratio of 30:1. To reduce the influence of out-of-plane shear computation, a large span–thickness ratio was selected. Failure in all samples occurred under quasi-static conditions. The effect of fiber reinforcement on the flexural properties of GF/SF/CTS hybrid sandwich composites are shown in Figure 4. The average ultimate flexural strength and modulus of GF/SF/CTS hybrid sandwich composite scaffolds were 260 ± 6.47 MPa and 18.35 ± 0.25 GPa, respectively. The flexural properties of sandwich composite scaffold samples are tabulated in Table 3. 

**Figure 5:**
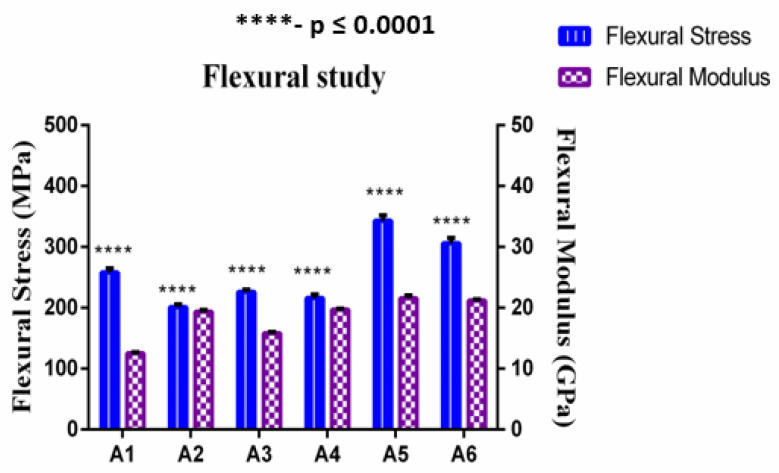
Flexural modulus vs. flexural strength of GF/SF/CTS composite scaffolds. Data are represented as means of triplicate (n = 3) ± SD, where **** indicates *p* ≤ 0.0001.

Figure 4 shows that the addition of CTS particles enhanced the flexural strength of composite scaffolds. The coarse surface on the fiber also enhanced the interaction ratio between the matrix and fiber, which helps in improving the mechanical interlocking capacity between the matrix and fiber. Figure 5 shows that the A1 and A5 composites have high flexural strength of 258 and 343 MPa, respectively, compared to that of the A3 composite scaffolds (226 MPa) due to the random dispersal of CTS. Whereas, due to eventual distribution of chitosan in A5 composites, flexural properties improved drastically. The flexural strength of three-layer SF scaffolds in A1 and A5 were 12.40% and 34.11%, showing a more significant difference (****) (*p* value ≤ 0.0001) than A3 composite scaffolds. Similarly, the flexural strength of four-layer SF scaffolds in A4 and A6 were 6.9% and 34%, showing a more significant difference (****) (*p* value ≤ 0.0001) than A2 composite scaffolds.

**Figure 6:**
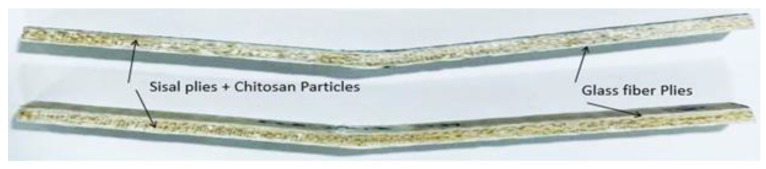
GF/SF/CTS hybrid sandwich composite scaffolds in a three-point bending.

All the GF/SF/CTS composite scaffolds failed at the outer surface (GF) followed by a progressive delamination and buckling of SF layers. The typical failed GF/SF/CTS hybrid sandwich composite scaffolds under three-point bending is shown in Figure 6.

### 4.3. Compressive Properties of the Composite Scaffolds

**Figure 7:**
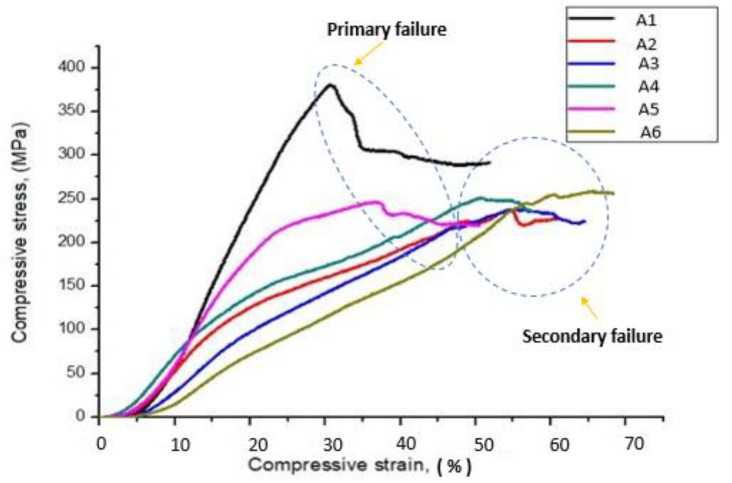
Stress Vs. strain variation of GF/SF/CTS composite scaffolds under compressive test.

The capability of a material to resist breaking under compression stress is one of the significant properties for bone plate application. The stress–strain curve related to the compressive behavior of composites is revealed in Figure 7. The figure shows that the addition of CTS particles enhanced the compressive strength of composite scaffolds and A5 composites shows higher compressive strength compared to the other combinations.

**Figure 8:**
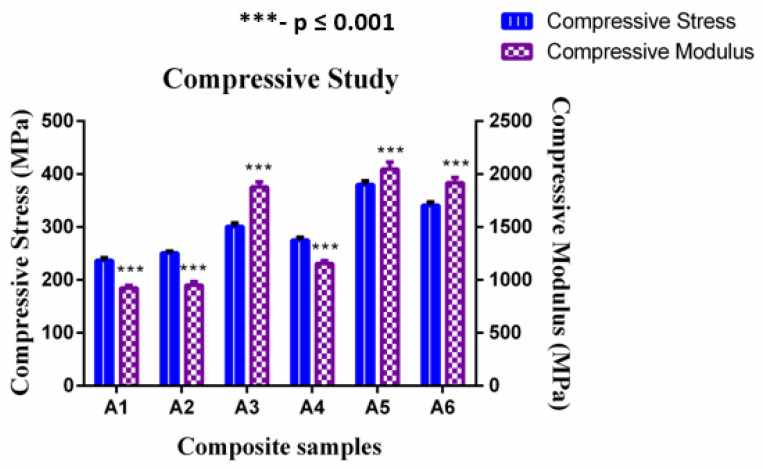
Compressive modulus vs. compressive strength of GF/SF/CTS composite scaffolds. Data are represented as means of triplicate (n = 3) ± SD, where *** indicates *p* ≤ 0.001.

The compressive strength of A3 and A5 composites were 21.26% and 37.63% higher than that of A1 composites. This clearly indicates that the inclusion of CTS particles improves the compressive strength of composite scaffolds. Similar trends were observed in A2, A4, and A6 composites. The compressive strength of A4 and A6 composites were 8.72% and 19.35% higher than that of A2 composites. Overall, a three-sisal core layer of A5 composite exhibits a more significant difference (***—*p* value ≤ 0.001) of compressive strength as compared to the four-sisal core layer of A6 composites as shown in Figure 8. The presence of more SF layer in the composite structure improves the brittleness of the structure, which leads to early failure at lower loads [25]. The average compressive strength and compressive modulus of GF/SF/CTS hybrid composite scaffolds were 297 ± 5.57 and 1479 ± 41.83 MPa, respectively. 

### 4.4. Micro Hardness Behavior of Composite Scaffolds

Vickers micro hardness values for the GF/SF/CTS composite scaffold samples are shown in Table 4.

**Figure 9:**
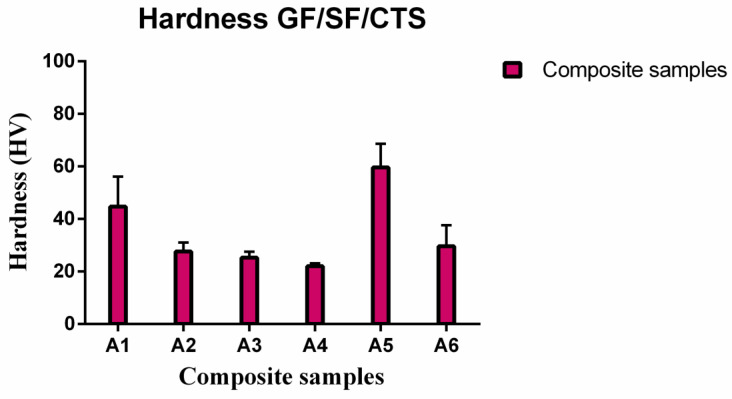
Micro hardness of GF/SF/CTS composite scaffolds.

Table 4 shows that the micro hardness of three-layer sisal core sandwich composites (A1 and A5) increased from 44.7 to 59.6 HV after the addition of CTS. The maximum Vickers hardness of 59.6 HV was obtained at 2 wt% CTS in the three-layer sisal core sandwich composites (A5 composites). A similar trend was observed in the four-layer sisal core sandwich composites as the maximum micro hardness value was 29.6 HV with the addition of 2 wt% of CTS (A6 composites). However, A5 composites show the highest micro hardness value among the composites (Figure 9) due to the uniform dispersal of CTS in the composites. The four-layer SF core sandwich composite samples (A2, A4, and A6) show an overall decreasing hardness values due to the flexible nature of SF (Figure 9).

### 4.5. Surface Morphology Study

**Figure 10:**
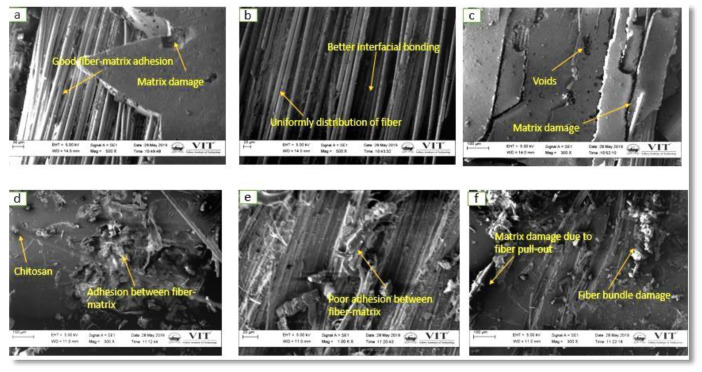
Tensile fractured surface of different composite: (**a**) A1 (**b**) A2 (**c**) A3 (**d**) A4 (**e**) A5, and (**f**) A6.

**Figure 11:**
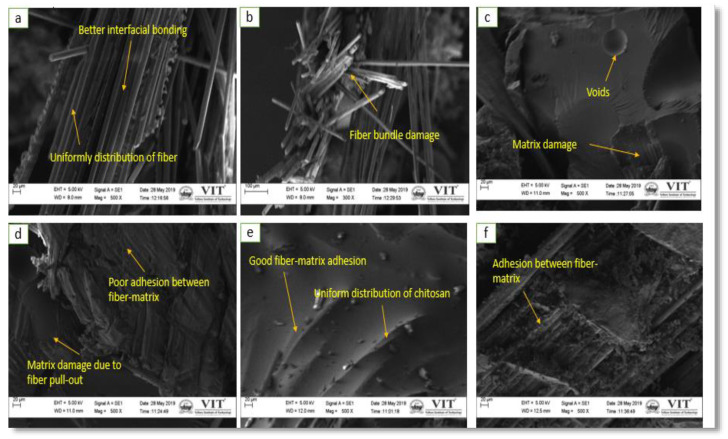
Flexural fractured surface of different composite: (**a**) A1, (**b**) A2, (**c**) A3, (**d**) A4, (**e**) A5, and (**f**) A6 composites.

**Figure 12:**
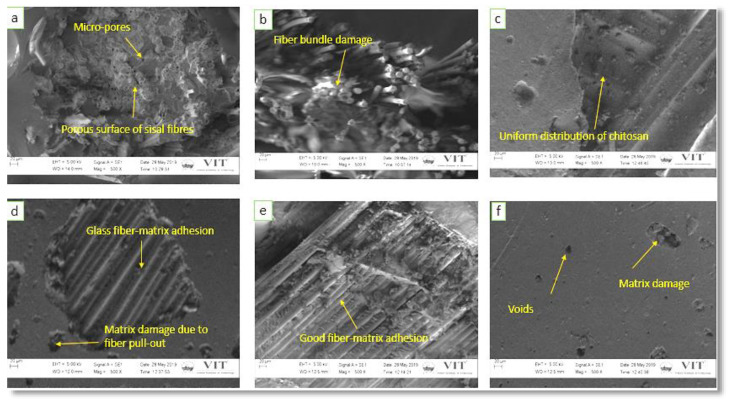
Compressive fractured surface of different composite used in this study: (**a**) A1, (**b**) A2, (**c**) A3, (**d**) A4, (**e**) A5, and (**f**) A6 composites.

Morphological investigations on the fractured tensile, flexural, and compressive fractured test samples were analyzed using SEM micrographs to understand the failure mechanism. For a better understanding, the morphological behavior has been studied with the low magnification (300× and 500×) for Figure 10a,c,d,f and a high magnification (1k×) for Figure 10e. However, for images in Figure 11 and Figure 12, consistent magnification of 500× was used.

Figure 10b,d show the uniform distribution of fiber and CTS particles in the composite structure. It is evident that failure took place in the bend direction due to the longitudinal tensile load and this assures the presence of good interfacial connection between the matrix and the fiber. No crack was observed in the A2 and A4 composite scaffolds, therefore A2 and A4 composites offers superior mechanical properties related to other combinations. Matrix damage and voids were present in the A1 and A3 composites due to their poor load-carrying capacity under longitudinal direction. Due to the random distribution of CTS and fibers in the matrix, the stress was distributed unevenly which leads to the development of internal defects in the A5 and A6 composite structures [28].

Figure 11a displays evident uniform stress distribution between the fiber and matrix under flexural load in A1 composites. This is because the A1 composites shows superior flexural properties compared to the A2 and A3 composites. Figure 11b shows fiber bundle damage in the A2 composites, which reduced the flexural properties. Figure 11c,d show that A3 and A4 composites have lower flexural strength, because of the existence of voids in the composite structure, which leads to matrix destruction during flexural loading and inadequate adhesion between the fiber and matrix. Figure 11e,f show a uniform distribution of CTS particles in A5 and A6 composites, which confirms the presence of uniform matrix surface in A5 and A6 composites. Thus, A5 and A6 composites show greater flexural properties compared to the other combinations. It can be concluded that the addition of CTS at 2 wt% for three- and four-layer composites (A5, A6) enhances the flexural properties.

Figure 12a,b show that A1 and A2 composites have lower compressive strength compared to the other combinations because of the existence of voids, which leads to matrix destruction at the time of compressive loading. Figure 12c–f reveal that the addition of CTS particles improved the compressive properties of A3, A4, A5, and A6 composite scaffolds and the A5 composite shows higher compressive strength compared to other combinations.

### 4.6. Wettability

**Figure 13:**
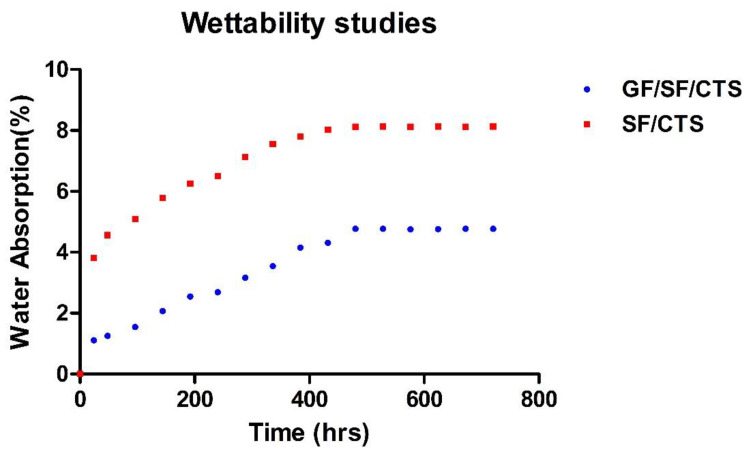
Water absorption of GF/SF/CTS and SF/CTS composites.

The water absorption behavior of the composite scaffolds is displayed in Figure 13. For comparison, water absorption tests were conducted on SF/CTS/epoxy composites and GF/SF/CTS composite for sample A5. The average water absorption percentage for SF/CTS/epoxy and optimized GF/SF/CTS composite sample A5 after 720 h were 6.953% and 3.436%, respectively. The GF/SF/CTS composite scaffolds possess lower water absorption rate compared to SF/CTS/epoxy composites. The water CA for SF/CTS/epoxy and GF/SF/CTS/epoxy were calculated as 54.28° ± 3.06° and 92.41° ± 1.71°, respectively, using the sessile drop technique. The SF/CTS/epoxy composite scaffolds exhibit a hydrophilic character, because the calculated CA value was less than 90°, whereas GF/SF/CTS/epoxy composite scaffolds shows a hydrophobic nature, with the CA value of more than 90°. 

### 4.7. Porosity of the Composite Scaffolds

Composite scaffolds porosity is known as the void space percentage in a composite structure. Pores are important for bone tissue formation. They permit the movement and propagation of osteoblast cells inside the scaffolds. Furthermore, porous surface of the scaffolds improves the mechanical interlocking and stability between the composite scaffolds and the surrounding natural bone. Researchers reported that lower porosity results in cell aggregation that develops osteogenesis; similarly, higher porosity develops bone ingrowth and diminished mechanical properties [53]. Researchers proved that more voids present in the composite structure develops non-uniform distribution of loads on the composite structure, which leads to reduced mechanical properties [54]. The porosity percentage of GF/SF/CTS composite scaffolds is shown in Table 5. The average porosity of GF/SF/CTS composites scaffolds was 3.243%. The result suggests that the GF/SF/CTS composite scaffold is a promising replacement biomaterial for bone plate fixation applications. 

## 5. Conclusions

Researchers have investigated the development of materials for orthopedic applications using the conventional materials such as stainless steel, titanium, cobalt and zirconium. However, these materials are much stiffer than human cortical bone, which results in the stress shielding effect. In order to reduce this stress shielding effect hybrid polymer composite can be used. In this study, GF/SF/CTS reinforced polymer composite scaffolds were produced by vacuum bag method.

- The mechanical properties of the GF/SF/CTS composite scaffolds reveal that the flexural stress (260 ± 6.47 MPa) and compressive stress (297 ± 5.57 MPa) were higher compared to those of the tension (134 ± 3.7 MPa).
- The hybrid GF/SF/CTS composite sample A5 showed considerable improvements in a compressive strength of 380 MPa (37.63%) and flexural strength of 343 MPa (34.11%) compared to other combinations.
- The hybrid GF/SF/CTS composite sample A5 showed maximum Vickers hardness of 59.6 ± 9.0 **HV** value compared to other combinations.
- The wettability study results reveal that the water CA of GF/SF/CTS composite scaffolds as 92°, exhibiting a hydrophobic nature. Whereas, the SF/CTS composite shows a hydrophilic nature at CA 57°.
- Water absorption properties show that the GF/SF/CTS composite scaffolds have less water absorption rate of 3.436% compared to the SF/CTS composites 6.953%.
- The GF/SF/CTS composite scaffolds showed considerable average porosity percentage of 3.243% using the ethanol infiltration method. The optimum value of porosity allows the growth of cells inside the scaffolds and thereby improving the mechanical stability between the composite scaffolds and natural bone.
- The statistical analysis showed that the null hypothesis is invalid and there are statistically significant differences between the mechanical properties and composite configurations.

The GF/SF/CTS composite scaffolds sample A5 exhibited a coarse morphology with significant mechanical properties, and less water absorption for a substantial period of time. The higher mechanical properties are due to the “sandwich structure effect” of GF/SF/CTS composite scaffolds. In addition, the failure stress in both bending and tension has been sufficiently strong to bear clinical-type forces as occurred in bone fracture plates. Such a hybrid sandwich structure makes an ideal candidate for designing optimized structure for femur bone plate applications. Based on the previous results reported on the hybrid composites (Refer Table 6), the GF/SF/CTS composite scaffolds showed better mechanical properties and it can be used as a promising biomaterial for orthopedic bone plate fracture fixation. 

In future, these composite scaffolds can be further used to conduct biocompatibility tests such as hemocompatibility, “in vitro” stability, and cytotoxicity to ensure that the fabricated hybrid composite will not cause unwanted biological reactions with the human body.

## Figures and Tables

**Table 1:** Work reported on stacking sequence of hybrid polymer composites.

Natural/Synthetic Hybrid Fiber	Polymer Matrix	Stacking Sequence/Properties	Applications	References
Flax (F)/carbon fiber (C)	Epoxy	2C-16F-2C/ high ultimate strength in both tension and flexural loading	Tissue engineering applications	Bazheri et al. (2013) [42]
Hemp (H)/jute (J)/sisal fiber (S)	Epoxy	SSJHJHSS/ Higher tensile, flexural and compression	Femur bone applications	Gouda et al. (2014) [33]
Flax (F)/glass fiber (G)	Epoxy	2G-12F-2G/ Higher stiffness and strength in bending	Femur bone plate applications	Manteghi et al. (2017) [34]
Flax (F)/glass fiber (G)	Epoxy	GGFFFFGG/ Higher bending modulus and lower water absorption rate	Biomedical applications	Cheour et al. (2020) [44]

**Table 2:** Compositions and design of sample composites.

Sample Designation	Chitosan (wt%)	Sisal Fiber (Layer)	Glass Fiber (Layer)
A1	0	3	2
A2	0	4	2
A3	1	3	2
A4	1	4	2
A5	2	3	2
A6	2	4	2

**Table 3:** Mechanical properties of GF/SF/CTS composite scaffolds.

Composite Samples	Tensile Stress (MPa)	Tensile Modulus (MPa)	Flexural Stress (MPa)	Flexural Modulus (GPa)	Compressive Stress (MPa)	Compressive Modulus (MPa)
A1	137 ± 7.08	5698 ± 194	258 ± 6.87	12.511 ± 0.18	237 ± 4.72	925 ± 25
A2	146 ± 5.50	6341 ± 129	201 ± 4.43	19.373 ± 0.26	251 ± 3.27	952 ± 32
A3	135 ± 3.01	5665 ± 178	226 ± 3.89	15.813 ± 0.23	301 ± 6.58	1879 ± 48
A4	139 ± 1.33	6646 ± 146	216 ± 5.91	19.705 ± 0.16	275 ± 5.42	1155 ± 27
A5	120 ± 1.09	6576 ± 153	343 ± 9.02	21.561 ± 0.47	380 ± 7.12	2046 ± 68
A6	131 ± 4.42	6054 ± 186	306 ± 8.71	21.180 ± 0.21	341 ± 6.36	1917 ± 51

**Table 4:** Micro hardness properties of GF/SF/CTS composite scaffolds in terms of mean ± SD.

Composite Samples	Vickers Micro Hardness (HV)
A1	44.7 ± 11.4
A3	25.3 ± 2.2
A5	59.6 ± 9.0
A2	27.6 ± 3.4
A4	22 ± 1.1
A6	29.6 ± 8.0

**Table 5:** Porosity percentage of GF/SF/CTS composite scaffolds.

Composite Samples	Initial Weight (g)	Final Weight (g)	Percentage of Porosity (%)
A1	0.6994	0.7247	5.87
A2	1.2798	1.3021	3.10
A3	0.6756	0.6970	4.96
A4	0.8510	0.8600	1.27
A5	1.3376	1.3558	2.679
A6	1.4679	1.4810	1.5821

**Table 6:** Mechanical properties of reported work on hybrid composites for orthopedic applications.

Material	Tensile Strength (MPa)	Bending Strength (MPa)	Compressive Strength (MPa)	References
Sisal/carbon fiber/polyester	84.44–107.51	140.89–169.14	-	Khanam et al. (2010) [27]
Sisal/banana/roselle	60.04–72.41	102.41–118.51	-	Bharanichandar et al. (2014) [45]
Femur bone	43.44 ± 3.62	84.04 ± 9.91	115.29 ± 12.94	Gouda et al. (2014) [33]
Current GF/SF/CTS	120–146 *	201–343 *	237–380 *	-
